# Male mating preference in an ixodid tick

**DOI:** 10.1186/s13071-022-05419-z

**Published:** 2022-09-07

**Authors:** Gerardo Fracasso, Dieter Heylen, Erik Matthysen

**Affiliations:** 1 Evolutionary Ecology Group, Department of Biology, University of Antwerp, 2610 Wilrijk, Belgium; 2 Eco-Epidemiology Group, Department of Biomedical Sciences, Institute of Tropical Medicine, 2000 Antwerp, Belgium; 3 Interuniversity Institute for Biostatistics and Statistical Bioinformatics, Hasselt University, 3590 Diepenbeek, Belgium

**Keywords:** *Ixodes arboricola*, Tree-hole tick, Male mate choice, Sexual selection, Mating strategy, Chemical communication

## Abstract

**Background:**

Mate choice is a fundamental element of sexual selection and has the potential to shape the evolution of traits. Mate choice based on body size has been shown to be a common trait in several arthropod species. In hard ticks, a taxon of medical and veterinary importance, engorgement weight is positively correlated with reproductive output but it is unknown whether adult males show mate choice. Here, we experimentally investigated whether males (i) use chemical cues to choose their mating partner, (ii) consistently choose for the same female individual and (iii) prefer females with highest weight after feeding.

**Methods:**

We used two experimental setups which allowed chemical communication between ticks: (i) a horizontal tube preventing physical contact with the female and (ii) an arena where tactile cues were allowed. In total, we tested 62 different triads in 124 tests (66 tests in the horizontal tube and 58 in the arena) composed of one male that could choose between two engorged females. Specifically, we tested 42 triads in the tube and 46 in the arena; 24 triads were repeatedly tested in the tube while 38 triads were tested in both setups.

**Results:**

We found no preference for individual or heavier females in either setup. However, in the horizontal tube setup, males significantly preferred females that were not visited by them in the previous test.

**Conclusions:**

Our results suggest a lack of male mate choice despite heavier females having higher fecundity. However, future studies should take into account that males may recognize the potential mating partners they previously met.

**Graphical Abstract:**

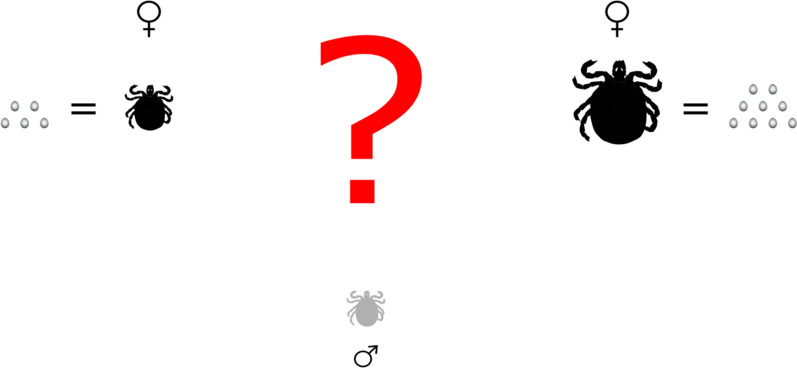

## Background

Sexual selection is among the most important evolutionary processes shaping the morphology, behaviour, life history, and ecology of species [1, 2]. One of the main components of sexual selection is mate choice, i.e. the differential sexual response leading to non-random mating with respect to one or more traits that are displayed in sexually mature individuals of the opposite sex and same species. Mate choice alters the reproductive success of individuals [3–6] and can evolve through direct and indirect selection on mating preferences. The evolution of mate choice usually occurs in the sex with greater reproductive investment, either the one with greater parental care or that with higher mating effort [7–9]. The evolution of mate choice is influenced by several factors that include — but are not limited to — mating investment, operational sex ratios (i.e. proportion of sexually active males and females), costs and benefits of choosiness and variation in mate quality [7, 10, 11]. For example, mate choice is favoured when the number of available mates is higher than the capacity to mate [7]. This condition is often satisfied when there is a simultaneous or frequent encountering of potential mating partners [12, 13]. High investment in mating and high variance in the quality of mating partners are also important promoters of mate choice. Several traits can be used to assess mate quality in the context of mate choice. Specifically, a trait used for mate assessment needs to satisfy three criteria [14]: (i) its expression in the chosen mate influences the fitness of the individual making the choice; (ii) there is considerable variation between potential mates for such trait; and (iii) the trait (or a correlated one) can be reliably evaluated prior to mating.

Male mate choice was first proposed to occur in species with reversed sex roles, i.e. in species where males exhibit higher parental care and females compete for them [15]. However, theoretical studies suggest that male mate choice may occur and evolve under broader conditions than originally thought, such as in the absence of male parental care, in the presence of more sexually active females than males (i.e. female-biased operational sex ratio) or in polygynous species [7, 16].

Arthropods are an abundant and taxonomically diverse group of organisms for which mate choice has been observed in multiple taxa, including insects [10, 17], arachnids [18, 19] and crustaceans [20]. In this taxon, body size is considered to be one of the most prevalent choice criteria [10, 21, 22]. Among arthropods, ticks are haematophagous ectoparasites that transmit a large number of diseases [23–25], but little is known on their mating strategies. Since mate choice can play an important role in selecting for a vast array of traits [1], understanding if and how ticks choose their mating partners will help us to comprehend how tick traits can have evolved and diverged between species. In hard ticks, mating can occur both before (preprandial) and after (postprandial) feeding. However, evidence shows that in some tick species males are more attracted to adult females that are engorging (e.g. in *Ixodes ricinus*), or fully engorged (e.g. in *Ixodes arboricola*) [26, 27]. By mating with engorged females, males avoid the risk of reproducing with ticks that could then fail to find a host and feed. Body size and engorgement status may be assessed through chemical or tactile cues. Although pheromones have not (yet) been clearly identified in ixodid ticks, chemical cues have been shown to play an important role in tick mate finding behaviour [28–30]. Visual capabilities beyond the perception of day-night cycle are to be excluded as only few bilaterally arranged photoreceptors have been found in a congeneric tick [31].

Some life-history characteristics suggest that mating is costly for males of the genus *Ixodes*. For instance, experimental evidence suggests that prostriate males, contrary to other tick groups, may only be able to inseminate a few adult females [33–35]. Moreover, prostriate ticks often remain in copula much longer than the time required for sperm transfer [26, 33, 34]. This form of mate guarding is likely an adaptation to prevent insemination from other males, thus ensuring paternity of the offspring [36]. However, postcopulatory mate guarding is costly for males as during this period further reproduction events are prevented. Moreover, mate guarding can incur additional costs if a female of poor quality is chosen as mating partner while more fecund females are being fertilized by other males. In these conditions, it can be hypothesized that male mate choice will be favoured by selection.

Ticks are an excellent model system for the study of host-parasite interactions at the individual level [37, 38]. Nevertheless, our knowledge about mate choice in this group of ectoparasites is scant. Moreover, evidence shows that different tick-borne diseases, such as the Lyme disease caused by *Borrelia* spp. [39], the tick-borne encephalitis virus [40] and Rickettsiae [41] can be sexually transmitted in ticks. Hence, understanding tick mating behaviour may also improve our comprehension of the population and evolutionary dynamics of ticks and the pathogens they vector.

The tree-hole tick *Ixodes arboricola* Schulze and Schlottke [42] is a bird-specialized nidicolous tick that lives in tree holes and nest boxes. Immature stages feed throughout the year, mainly on adult birds, while adult females feed on nestlings [43]. Several *I. arboricola* characteristics suggest that male mate choice should be favoured in this species. First, this tick has a female-biased sex ratio [44] whereby adult females outnumber adult males, thus promoting male choice. Second, since adult *I. arboricola* females almost exclusively engorge on nestlings during the breeding season [43], they synchronize their attachment with host development [45]. In the hosts exploited by the tree-hole tick, all nestlings grow at the same time in the nest. Hence, every year the majority of tick females likely engorge and detach within a couple of days [46] making them available for mating almost simultaneously and providing the few males with the choice between several females. At this stage females can be fertilized and subsequently lay eggs. Males that initiate mating in late May have a reduced number of potential partners from which to choose. Third, in *I. arboricola* engorgement weight is highly variable, and strongly positively correlated with the number of hatched eggs [26], similar to other tick species [47–49]. Sexually active males would thus gain fitness benefits from choosing the heaviest engorged female available. Fourth, males exhibit postcopulatory mate guarding behaviour even beyond egg deposition [26]. It has been suggested that this is an adaptive strategy to prevent fertilization from other males given the absence of sperm precedence [26]. Fifth, due to the female’s impaired mobility after feeding, females have little opportunities to refuse the mobile (unfed) male and its mating attempts. However, females could still influence male fertilization success through other physical or chemical mechanisms, i.e. cryptic female choice [50].

In this study we tested (i) whether chemical or tactile cues mediate information on mate choice; (ii) whether *I. arboricola* males are consistent in their preference for individual females; and (iii) if males prefer heavier females. To address these questions we used two different experimental setups.

## Methods

*Ixodes arboricola* is an endophilic hard tick (Ixodidae) distributed across the Palearctic region [51, 52]. It feeds primarily on cavity-nesting birds, in particular great tits *Parus major* and blue tits *Cyanistes caeruleus* [43, 53, 54].

Adult ticks were derived from a breeding laboratory population that was founded with ticks from four areas to ensure genetic variability (see [37] for details). The immature developmental stages (larvae and nymphs) fed on wild-caught adult great tits that were kept in captivity only for the duration of tick engorgement, while adult females fed on great tit nestlings in nest boxes during the great tit breeding season.

**Fig. 1:**
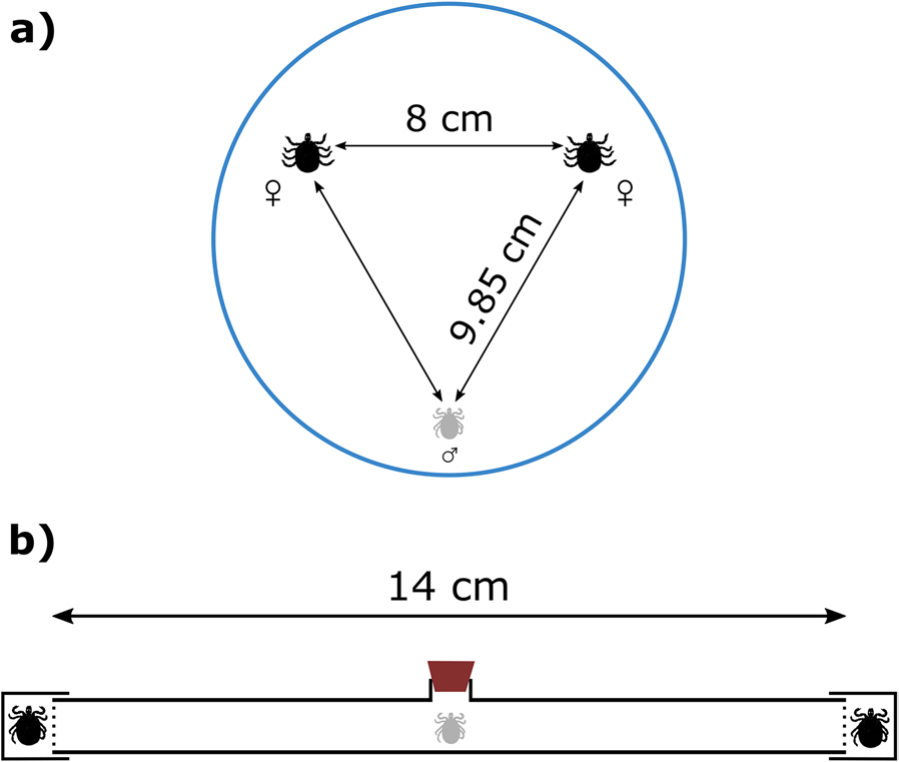
Overview of the study setups. One male (grey) was given the choice between two engorged females (black) in two different setups: an arena (**a**) and a horizontal tube (**b**). Females were weighed before the experiment and randomly assigned to a test. **a** In the arena setup, the two female ticks were put upside down, 8 cm apart from each other, while the male tick was placed at a distance of 9.85 cm from each female. A plastic dome (blue) prevented ticks from escaping, while keeping the arena free from uncontrolled airflow. **b** In the horizontal tube setup, female ticks were placed 14 cm apart, and a plastic mesh (dashed lines) prevented them from being visible to the male tick. A plastic cork (brown) sealed the entrance of the horizontal tube

In June 2019, male mating preference was tested in triads consisting of one adult male and two fully engorged female ticks. Females were weighed the day of collection, i.e. immediately after engorgement and before the experiment, and randomly assigned to a triad and position in the setup. No female had the opportunity to mate before the experiments. Mating preference was tested in two experimental setups with different characteristics (Fig. 1): (i) a horizontal tube and (ii) a circular arena. The two setups differed in the amount of tactile information available: females could be touched by the male in the arena but were physically not accessible to males in the horizontal tube (see following sections for details on experimental setups). In contrast, female chemical volatiles could always be perceived by the male in both setups although the arena had a much bigger air volume. The majority of triads, consisting of the same male tick and a couple of female ticks, were tested in both setups, thus allowing estimation of the consistency of male choice for individual females in the presence or absence of tactile cues (between setups). In the horizontal tube, a number of triads were also tested twice. Female body weights ranged from 18.56 to 72.88 mg. The average weight difference between females in a triad was 10.71 mg in the horizontal tube and 10.85 mg in the arena (range in both cases: 0.49–35.56 mg). Before every trial, the experimental setups were rinsed with 70% ethanol and left to dry. Gloves were worn by the experimenter who was blind to tick characteristics and previous male choice. Experiments were carried out under low light conditions to mimic natural conditions. The experimental room was air-ventilated, thus reducing any odour contamination between trials and experimental setups.

### Horizontal tube setup

The horizontal tube setup allows the male to only use chemical cues to assess potential mating partners. Females were placed at opposite ends of a 14-cm-long circular glass tube (diameter: 18 mm) (Fig. 1). A piece of mesh cloth on each side held the female in place, preventing physical contact with the male as well as actual mating. Both ends of the tube were closed off with a plastic lid, thus preventing unidirectional airflow within the tube. After both females were placed in position, a male was put in the centre of the glass tube through a hole subsequently closed off with a plastic cork. Males were positioned perpendicularly to the females, and their position was recorded at 1-min intervals for a total of 25 min. A female was considered chosen if the male crawled within 1.5 cm of it. The same tube was used for all tests, and its position relative to the observer and the room was kept constant throughout the experiment. Mesh cloths and the plastic cork were always rinsed in 70% ethanol and randomly swapped between trials. The total time spent in each arm of the maze was also recorded. Males were chosen not to be siblings of any of the two tested females. To estimate repeatability of male choice within the experimental setup, triads were tested a second time 1–2 days later (median: 2 days) after swapping the females’ positions. Males did not show any significant preference for either side of the tube in the first or second tube test (both *P* > 0.175).

### Arena setup

In the arena setup, both tactile and chemical stimuli could be used to assess the mate. A circle of 12-cm diameter on the smooth plastic surface of a table delimited the setup. Inside this circle, two females were placed 8 cm apart from each other leaning on the dorsum to prevent them from moving in the arena. The male was placed at a distance of 9.85 cm from each of the female ticks (Fig. 1). In this way, we maximized the distance between the male and the two females below the transparent plastic dome (placed over the arena) to prevent a unidirectional airflow while at the same time allowing the male to approach the females from any direction. A female was considered chosen when the male crawled on top of it. Each trial ended when a female was chosen or after 50 min in case of no choice.

In the arena setup, males could initiate copulation soon after female choice, and this could affect subsequent mate choice. Therefore, all triads undergoing both experimental setups were first tested in the horizontal tube followed a few days later by the arena test (average time after the last tube test: 5 days [range: 4–8 days]). All females that could have been fertilized in the arena as a result of male choice were not tested a second time.

In total, we carried out 124 tests investigating 62 different triads with a total of 62 males and 93 different females. Thus, some males and females were tested multiple times to estimate choice repeatability within setups and choice consistency between setups. In the horizontal tube setup, 42 triads were tested, of which 24 were repeated once, giving a total of 66 tube tests. In the arena setup, we carried out 58 tests in total, of which 12 consisted of only females that were not chosen in a previous arena test to estimate choice consistency across males. We tested 38 triads in both setups (see Table 1 for an overview).

**Table 1:** Sample size of the individuals and tests carried out in the horizontal tube, in the arena, and in both experimental setups

Sample size and tests	Horizontal tube setup	Arena setup	Both setups
*Individuals*			
Females	84	92	76
Males	42	58	38
*Tests*			
Tested triads	42 (35)	46 (29)	16
Repeated triads	24 (21)	12^a^ (5)	22
Total tests	66 (56)	58 (34)	38

We tested the choice of every male between two different females, with every combination of male and females referred to as a triad

The number given in parentheses is the number of tests in which a choice was made by the male

^a^Triads tested with a different male

### Statistical analyses

Female tick weight was individually measured twice to the nearest 10^–2^ mg (scale model XS205; Mettler Toledo, Columbus, OH, USA), and the average value was used in the analyses. Analyses were carried out in R version 4.1.2 [55]. Preference for each female was binarily coded. Generalized linear models (logistic regression) were used to analyse differences in engorgement weight between chosen and not-chosen females. For every test, male choice was set as response variable while the weight difference between ticks was set as an explanatory variable. To avoid pseudo-replication, separate models were calculated for every setup and order of test, i.e. first or second test, unless we specifically tested repeatability or consistency between setups. Male mating repeatability was analysed by comparing the proportions of males that chose the same or the opposite female using a two-sample Z-test for equality of proportions with continuity correction, using only males that made a choice in both tests. Since the analysis of consistency between setups had a relatively low sample size, we used the binomial exact test instead of the Chi-squared test (*χ*^2^) as it is considered a better test at low sample sizes [56].

## Results

**Fig. 2:**
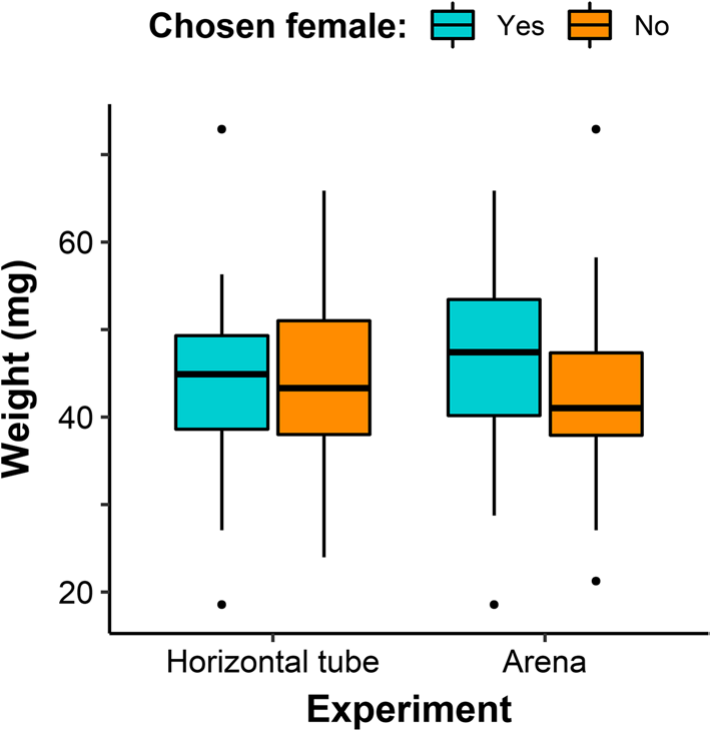
Difference in engorgement weight between chosen (light blue) and not-chosen (orange) adult female ticks in the horizontal tube setup (*N* = 35) and in the arena setup (*N* = 29) setup. Engorgement weight median (horizontal line), first and third interquartile range (box limits), minimum and maximum values (vertical lines) and potential outliers (dots) for both groups of females are shown.

When analysing only the triads tested for the first time, chosen females tended to be slightly heavier than non-chosen females both in the horizontal tube setup (median: 44.87 vs 43.30 mg) and in the arena setup (median: 47.40 vs 41.02 mg), but weight difference was not a significant predictor of male choice (tube: glm estimate = 0.00, *df* = 33, *P* = 0.77; arena: glm estimate = 0.00, *df* = 27, *P* = 0.27; Fig. 2). Results were similar when we considered all tests, i.e. including the repeated ones (tube: glm estimate = 0.00, *df* = 54, *P* = 0.92; arena: glm estimate = 0.00, *df* = 32, *P* = 0.33).

When testing the repeatability of male choice in the absence of tactile cues (horizontal tube), 5 males chose the same female (21%), 14 chose the opposite one (58%), and 5 more males did not make a choice in at least one of the two tests (21%).

Hence, males significantly preferred the opposite female (or the same side of the tube) during the repeatability test (Chi-square test, ${\chi }^{2}$ = 6.737, *df* = 1, *P* = 0.009; excluding the males that made only one choice). Consistency of male mate choice between the tube and arena setups was not significant, as nine males chose the same female, 10 chose the opposite one and 19 made no choice (Chi-square test, ${\chi }^{2}$ = 0, *df* = 1, *P* = 1).

In the horizontal tube, males spent more time (on average 17 vs 6 min) in the arm of the tube corresponding to the female they subsequently chose (Wilcoxon rank sum test, *W* = 409.5, *P* < 0.001), but no significant difference was found between heavier and lighter females (Wilcoxon rank sum test, *W* = 1289, *P* = 0.10).

## Discussion

Our results show that *I. arboricola* males do not show any preference for heavier engorged females although these are expected to produce more offspring [26, 37, 47, 48]. This lack of male mate choice for body size occurred both in the absence and presence of tactile cues. Very few observations on mate choice are available for other tick species. For example, in a population of *Dermacentor andersoni* polymorphic for body size (bimodal distribution), small males mated more frequently with large females but large males mated equally frequently with large and small mating partners [57]. Although we did not account for male weight, *I. arboricola* males are monomorphic for body size and hence less likely to show differences in preference between bigger and smaller males. In the wild, genotype analyses of *I. ricinus* showed evidence of assortative mating, a potential sign of mate choice, although the factors driving it are unclear [58].

A number of non-mutually exclusive hypotheses may explain the absence of male choice. First, males may have been unable to correctly assess female size. It is unknown to what extent this tick species relies on chemical, tactile or other cues to find and assess potential mating partners. Alternatively, the benefit of choosing between females may be lower than the cost of female assessment. It is worth mentioning that the accuracy of the prediction on the quality of a potential mate plays an important role in determining such costs.

Moreover, the chance for engorged ixodid females to avoid mating is small due to their impaired mobility. Ixodid females may thus greatly benefit from operating cryptic choice of male sperm after insemination has occurred [59], potentially hampering the evolution of male mate choice. Although cryptic female choice has been extensively documented in arthropods [60], very little is known about cryptic female choice in ticks.

Finally, our experimental males were kept in individual vials until testing. Hypothetically, the prolonged lack of females may have induced males to estimate that the risk of not mating was high. Males might thus have responded to these environmental conditions by reducing their choice behaviour in order to mate as quickly as possible. Plasticity in mate choice has been shown to vary with environmental and social conditions as well as with the chooser’s characteristics in many animal species [61]. In particular, both game-theoretical models and experimental evidence in arthropods have shown density-dependent plasticity in mate choice [11, 62, 63]. We suggest that future studies should assess whether tick mating behaviour is affected by the presence of potential mating partners.

Interestingly, *I. arboricola* males that were tested twice in the same setup (horizontal tube) showed a preference for females that were not previously visited, instead of repeating their choice. One hypothesis is that males may have recognized the previously encountered females and have avoided the one with whom they were previously unsuccessful. This hypothesis assumes that males somewhat remember their previous mating partners for at least a couple of days. Such memory could be achieved, for example, through chemosensory recognition. A wide range of arthropods has been shown to use cuticular hydrocarbons as recognition cues during mating and to utilize chemosensory self-referencing to identify recent mates, thus requiring minimal cognitive abilities [64]. A limitation of our setup is that it cannot rule out confounding factors, such as male preference for a specific side of the tube (which always had the same position with respect to the observation room) due to the fact that female positions were not randomized in the second test but always alternated between the two ends of the tube. Nevertheless, we believe this hypothesis is unlikely since there was no general preference for one particular side of the tube. Similarly, it cannot be excluded that males may have preferred to move in the same direction of the tube where they had encountered a female in the previous test, i.e. use this as a cue for an environment where females can be found. Moreover, the relatively low sample size, the lack of a wide choice area and absence of a standardized airflow from every female to the male may have reduced our capability to measure male mate choice.

Our findings suggest that *I. arboricola* males can exert choice in the absence of tactile cues. Ideal candidates to convey such information are pheromones. Although sex pheromones have not (yet) been identified in ixodid ticks (but see [29]), a large number of volatile and non-volatile pheromones have been shown to be involved in several phases of the life cycle of Ixodidae [32, 65]. We hypothesize that *I. arboricola* males may use pheromones secreted by females to recognize them. The use of sex pheromones had previously been suggested by the authors of a number of other studies in *I. ricinus* [28, 66, 67], although there is no general consensus [68]. Interestingly, when triads tested in the tube were later tested in the arena, males did not show a preference for the opposite female. One explanation could be that the volume of air was much bigger in the arena setup and that pheromone gradients were much more diffuse than in a linear setup. Alternatively, the different result could lend support to explanations based on males remembering a specific location (i.e. the arm of the tube) than an individual female. In this case, carrying out additional tests in the absence of the females could help to shed more light on male tick behaviour.

## Conclusions

In conclusion, we suggest that *I. arboricola* males do not prefer to mate with heavier and thus more fecund engorged females in the wild, nor did we find evidence for consistent preference for individual females across trials. On the other hand, the outcomes suggest that males may recognize individual females. An important implication is that subsequent choice tests separated by only one or two days may not provide independent results, and that possible memory effects for specific locations have to be taken into account. More studies on the role of sexual selection in ticks and other arthropod vectors would not only be beneficial for a better understanding of their mating mechanisms but also for a better comprehension of the selective pressures acting on these parasites.

## Data Availability

All data generated or analysed during this study are included in this published article.
